# Switching to external flows: perturbations of developing vasculature within chicken chorioallantoic membrane†

**DOI:** 10.1039/d4lc00311j

**Published:** 2024-05-06

**Authors:** Prasanna Padmanaban, Danny van Galen, Nasim Salehi-Nik, Mariia Zakharova, Loes Segerink, Jeroen Rouwkema

**Affiliations:** a Vascularization Lab, Department of Biomechanical Engineering, Technical Medical Center, Faculty of Engineering Technology, University of Twente Enschede The Netherlands mailprasanna03@gmail.com j.rouwkema@utwente.nl; b BIOS Lab on Chip group, MESA+ Institute for Nanotechnology, Technical Medical Center, Max Planck Institute for Complex Fluid Dynamics, University of Twente Enschede The Netherlands

## Abstract

The impact of fluid flow shear stresses, generated by the movement of blood through vasculature, on the organization and maturation of vessels is widely recognized. Nevertheless, it remains uncertain whether external fluid flows outside of the vasculature in the surrounding tissue can similarly play a role in governing these processes. In this research, we introduce an innovative technique called superfusion-induced vascular steering (SIVS). SIVS involves the controlled imposition of external fluid flow patterns onto the vascularized chick chorioallantoic membrane (CAM), allowing us to observe how this impacts the organization of vascular networks. To investigate the concept of SIVS, we conducted superfusion experiments on the intact chick CAM cultured within an engineered eggshell system, using phosphate buffered saline (PBS). To capture and analyze the effects of superfusion, we employed a custom-built microscopy setup, enabling us to image both superfused and non-superfused regions within the developing CAM. This study provides valuable insights into the practical application of fluid superfusion within an *in vivo* context, shedding light on its significance for understanding tissue development and manipulation in an engineering setting.

## Abstract 2

Not only the flow of liquid through vasculature, but also external fluid flows can affect the organization and maturation of vessels.

## Graphical abstract

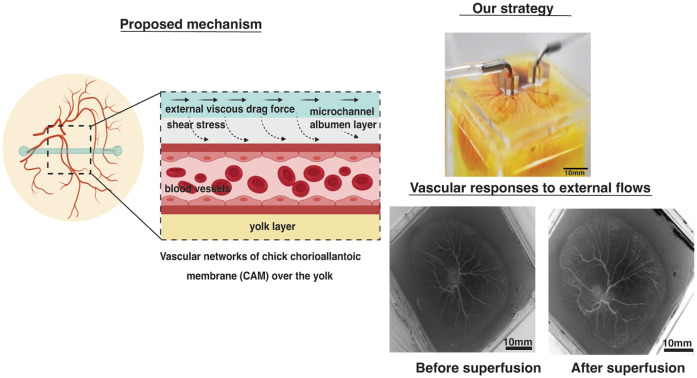

## Introduction

Physical transport mechanisms, including oxygen transport, nutrient distribution, and gas exchange facilitated by a flowing liquid within vascular networks and the surrounding interstitial spaces, are widely acknowledged as critical for tissue development and for shaping the overall architecture of vascular networks within tissues.^1,2^ These flow processes result in the generation of varying shear stresses within multi-sized vessels, which are governed by the pulsating heart in living organisms, as investigated using models such as chick,^3^ zebrafish,^4^ and frog embryos.^5^ Conversely, within developing vessels of engineered tissues, for instance, in microfluidic chips, these shear stresses are regulated by the pressure driven flows generated by micropumps. The former process involves complex flow patterns which are challenging to control, whereas the latter can be modulated by adjusting the flow rates or applied pressure gradients.

Complex flow patterns arising within intricate vascular geometries and interstitial tissue space include laminar, pulsatile,^6^ oscillatory,^7^ and turbulent profiles.^8^ It is known that fluid flow shear stresses arising from flows within vessels direct the organization and maturation of the vasculature.^9,10^ Previous studies have demonstrated that the development and organization of vascular networks and associated flows during embryogenesis can be altered through genetics and chemical treatments.^11,12^ Moreover, these vascular networks exhibit adaptability in response to hemodynamics and can be highly influenced by the environment.^9,13^ In native tissues, the endothelial cells that are lining the blood vessel walls are only exposed to fluid flow shear stresses on the luminal side of the vessel due to blood flow. Endothelial cells within an engineered platform such as microfluidic systems on the other hand, can be exposed to both internal luminal flows and external interstitial flows during vasculogenic and angiogenic processes. Even though there are clear indications that both internal and external flows can influence vascular organization *in vitro*, it has not been explored whether external fluid flows, which are generally easier to control in an engineering setting, can also influence vascular networks within a living organism. Therefore, to exert localized control over fluid flow induced shear stresses *in vivo*, we aim to explore methods that extend beyond genetic and chemical interventions and to actively direct the development and organization of vasculature using the support of controlled externally induced flows.

**Fig. 1:**
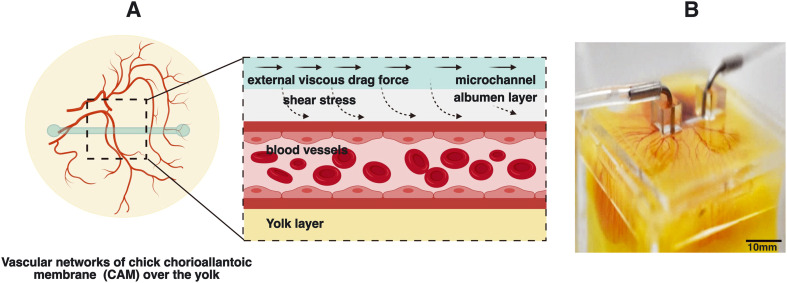
Superfusion induced vascular steering concept. (A) The illustration of external flow perturbations within developing vascular networks of the chick chorioallantoic membrane (CAM). (B) The experimental sample with microchannels integrated within the developing chick CAM cultured in the engineered eggshell system.

**Fig. 2:**
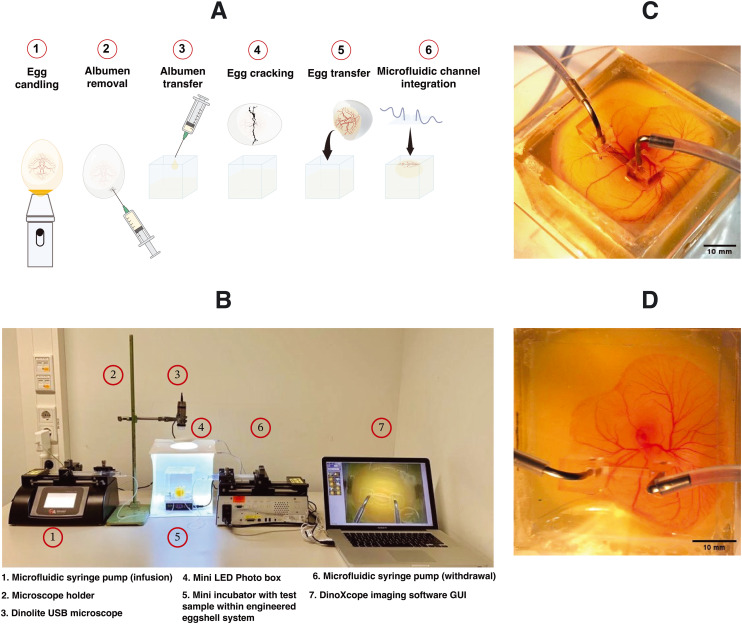
Experimental procedure and microscopy setup for *in vivo* superfusion. (A) An illustration of the experimental protocol for culturing the chick CAM using the engineered eggshell system and the incorporation of microfluidic channels. (B) The complete custom microscopy setup and the configuration of the microfluidic superfusion system. (C and D) The experimental test samples, showcasing the placement of microfluidic superfusion channels at various locations on top of the CAM tissue.

In this manuscript, we demonstrated an innovative strategy termed superfusion-induced vascular steering (SIVS), which involves the controlled application of external fluid flow patterns onto the vascularized CAM tissue (Fig. 1). We accomplished this by utilizing an engineered eggshell platform to culture the entire chick CAM specimens, which possess multi-sized vasculature networks (Video S3a†). Additionally, we integrated a perfusable microfluidic channel designed to facilitate the fluid flow of varying viscosities onto the surrounding microenvironment of the CAM tissue (Fig. 2C and D and Video S3b†). Using this methodology, we introduced changes to the CAM tissue by perfusing PBS and closely examining how this impacts the vascular organization in various regions of the CAM tissue (Fig. 4C and D, Videos S4a and b†). As a proof-of-concept test, we inserted a porous PDMS (polydimethylsiloxane) membrane into the microfluidic channel to further enhance the localized, controlled influence of fluidic shear stress on the adjacent microenvironment (Fig. 5A and B and S3 and Video S5†).

## Results

### Engineered eggshell system with an integrated microchannel for *in vivo* superfusion

The design of the engineered eggshell platform is based on a previously published system that has been adopted and re-engineered for the purpose of this study.^14^ The platform used comprises of two components: (1) a 2 mm thick polymethylmethacrylate (PMMA) frame that offers structural support and (2) thin PDMS membranes with a thickness between 500 and 700 μm, which are employed to create accessible windows as shown in Fig. 1B, S1 and S2.† To expedite and streamline the assembly of the eggshell, 3D drawing was generated using the “Lego-cuts” method through https://www.makercase.com. This approach allows for a quick and efficient construction of the eggshell. For the frame, 2 mm thick PMMA sheets were laser-cut and assembled, and quick-fix super glue (Loctite) was used to seal the gaps between the Lego-cut components, ensuring that no liquid leakage would occur. For the membrane containing the flow channel, PMMA rectangular blocks measuring 10 mm in length, 1 mm in width and 100 μm in height were laser-cut and affixed to square shaped PMMA sheets of 4 cm side length, to form a negative mold. The PDMS membrane was subsequently prepared by pouring the PDMS over the mold. After the assembly of the system, a 2 mm biopsy punch was used to create holes in the microchannel, establishing the inlet and outlet points for connecting the pump tubing as shown in Fig. 1B and 2C and D, respectively. The eggs were then introduced and cultured as shown in Fig. 2A. Using this methodology, chick embryos could be cultured successfully within the engineered eggshell system up to 4 days, after which experiments were terminated for ethical reasons. During this period, CAM development was comparable to *in ovo* controls.

### Computational fluid dynamics predictions of superfusion induced fluid flow shear stress within the CAM

**Fig. 3:**
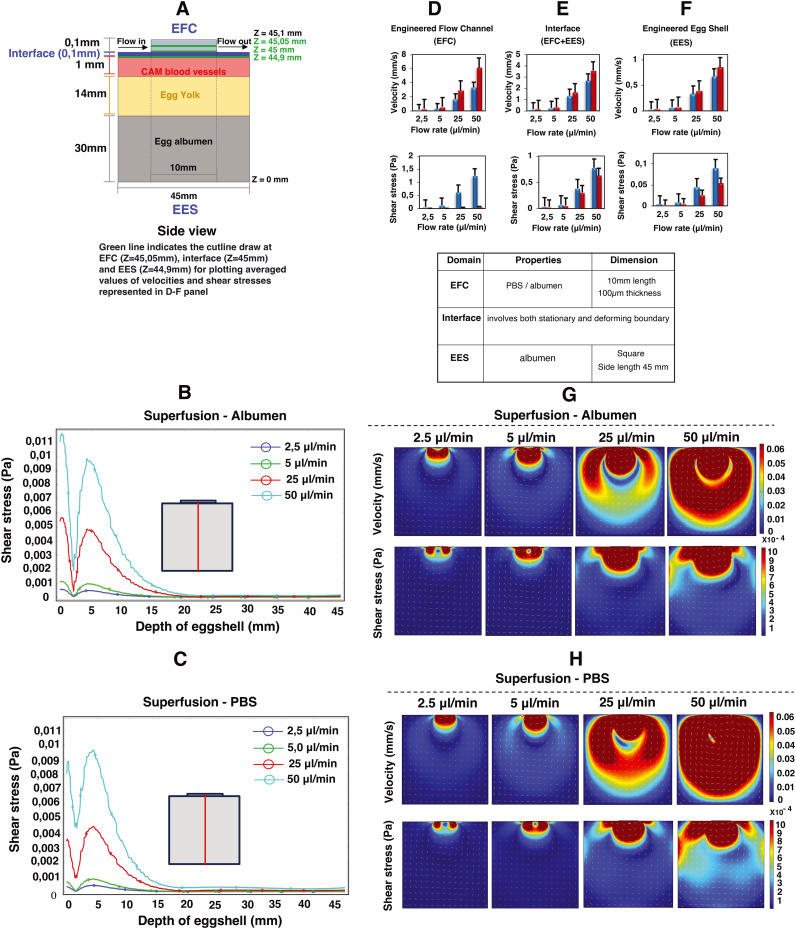
Impact of superfusion on shear stresses and velocities in the engineered eggshell system. This figure portrays 2D simulation data showcasing microfluidic superfusion in an engineered eggshell system. (A) An illustration of the 2D side view of the engineered eggshell, featuring the superfusion channel that served as the geometrical input for the simulation. It also outlines the locations of the microchannel, interface, CAM blood vessels, egg yolk and albumen, respectively. (B and C) The line plots of shear stresses along the depth of the engineered eggshell at increasing flow rates. The red line in the drawing highlights the location of a cut line made in the 2D model for analysis. (D–F) The averaged simulated values of velocities and shear stresses at three specific locations: the middle of the engineered flow channel (EFC) at *Z* = 45.05 mm, the surface of CAM blood vessels within the engineered eggshell (EES) at *Z* = 44.9 mm, and the interface between the EFC and EES at 45 mm from the bottom of the eggshell marked as *Z* = 0 respectively. This involves superfusing albumen and PBS fluids through integrated microchannels at progressively increasing flow rates. The three green lines in panel A indicate the locations of the cut lines made in the 2D model for generating the graphs. (G and H) The heatmaps of predicted velocities and shear stresses within the engineered eggshell upon albumen and PBS superfusion at increasing flow rates. Heatmaps were plotted using COMSOL Multiphysics software version 5.6 and graphs were plotted using Microsoft Excel software application.

Computational fluid dynamics (CFD) simulations were employed to estimate the superfusion induced velocities, pressure and shear stresses within engineered eggshell platforms containing an integrated flow channel. A 2D side view of the integrated microfluidic flow channel within the engineered eggshell, as shown in Fig. 3A, was designed as the geometric input for the CFD simulation using COMSOL Multiphysics software (version 5.6). Subsequently, the laminar flow physics module was selected to solve the Navier–Stokes equation, which helped to predict flow velocities, shear stresses, and pressure ranges that were shown as heatmaps in Fig. 3B–H.

Our assumptions for the 2D simulations aimed at modeling the mechanism of superfusion-induced vascular steering relied on a Newtonian model of albumen, despite its complex viscoelastic nature as albumen behaves both as a liquid and a gel with respect to temperature changes.^15^ Rheologically, the viscosity of albumen decreases with increasing shear stress.^16^ However, given that the shear stress values in the albumen are below 1 Pa as shown using the computational models, the changes in the rheological properties of the albumen upon superfusion are limited. Additionally, the rheological properties of albumen change during the embryonic development; the albumen viscosity in the embryonic development days EDD3 to EDD4 is lower compared to later development days, and hence the shear thinning effect is minimal. Meuer HJ and Egbers C (1990)^17^ emphasized that the egg yolk is encased in three concentric layers of albumen: an inner thin layer, a middle thick fibrous layer, and an outer thin layer. The thin albumen exhibits low viscosity, while the thick albumen demonstrates characteristics of high-viscosity yield stress liquids. They considered albumen as both Newtonian and non-Newtonian fluids. In the Newtonian case, the predicted albumen viscosities were found to be 4.6 mPa s and 4.9 mPa s for embryonic development days 0 and 4, respectively. Conversely, in the non-Newtonian case, the viscosities were 3.9 mPa s and 4.6 mPa s. Their respective yield stresses were noted as 22 μPa and 3.5 μPa for day 0 and day 4.^17^ As this study is performed during early development, the choice was made to model albumen with Newtonian properties to simplify the model. Hence, the elastic properties of albumen are ignored for model simplicity.^18,19^

The engineered eggshell (EES) domain consists of albumen, with the properties identical to the superfusion fluid except for the density and viscosity. The external boundaries were modeled as static no-slip boundary conditions, with the properties identical to the superfusion fluid except for the density and viscosity. Thus, our simulation does not include the outer PDMS and PMMA support components. The interface between the EES domain and the superfusion fluid in the microfluidic channel is also modeled as a no-slip boundary, which in this case means that the liquids at both sides of the interface have the same velocity.

Using a flow rate-driven model, at the entrance of the flow channel, inlet flow rates ranging from 0 to 50 μl min^−1^ were instituted, and a zero-pressure boundary was set at the outlet of the flow channel. The model was subjected to extremely refined physics-controlled mesh conditions consisting of ∼30 000 triangular elements. Two different perfusion liquids were simulated: albumen and PBS, represented by densities and viscosities *ρ*_albumen_ = 1035 kg m^−3^ and *η*_albumen_ = 0.0181 Pa s; *ρ*_PBS_ = 993 kg m^−3^ and *η*_PBS_ = 6.78 × 10^−4^ Pa s, respectively.^20,21^ As can be seen in Fig. 3, simulations reveal a direct linear correlation between albumen and PBS superfusion and generated shear stresses with rising flow rates. This unequivocally demonstrates that the viscous drag force generated at the interface (walls of the flow channel) directly perturb the CAM blood vessels positioned beneath, as illustrated in Fig. 3A.

Owing to the variance in fluid viscosities between albumen and PBS, higher flow rates during superfusion result in PBS flowing more rapidly than albumen. This leads to an increase in flow velocities and a decrease in shear stress levels, as predicted in Fig. 3D–H, in both the engineered flow channel and engineered eggshell compartments. Moreover, this outcome is reflected in the depth effect within the engineered eggshell, as displayed in Fig. 3B and C. As illustrated in Fig. 3A, G and H, the blood vessels within the CAM encounter higher shear stresses in comparison to the yolk and surrounding albumen. The higher shear stresses are attributed to the positioning of the flow channel directly over the blood vessels. The positioning of the flow channel on the top of blood vessels offers a distinct method for focusing the wide area of vasculature during superfusion. Conversely, Huang *et al.* demonstrated the perfusion's effect by choosing the side walls of the engineered eggshell.^22^ However, this side channel selection introduces variations in the sample, whereas our choice of placement on the top provides specific advantages in terms of blood vessel selection and control over imaging and incubator integration. It is crucial to recognize that due to egg size variations, embryo movement and yolk displacement during experiments, it is challenging to place the flow channel over a specific vessel for the duration of an experiment. As such, it is difficult to assess the effect of external flows on the remodeling of specific vessels or precise conserved vascular regions within multiple samples with the current setup.

### Superfusion flow conditioning alters the organization of developing vascular networks

**Fig. 4:**
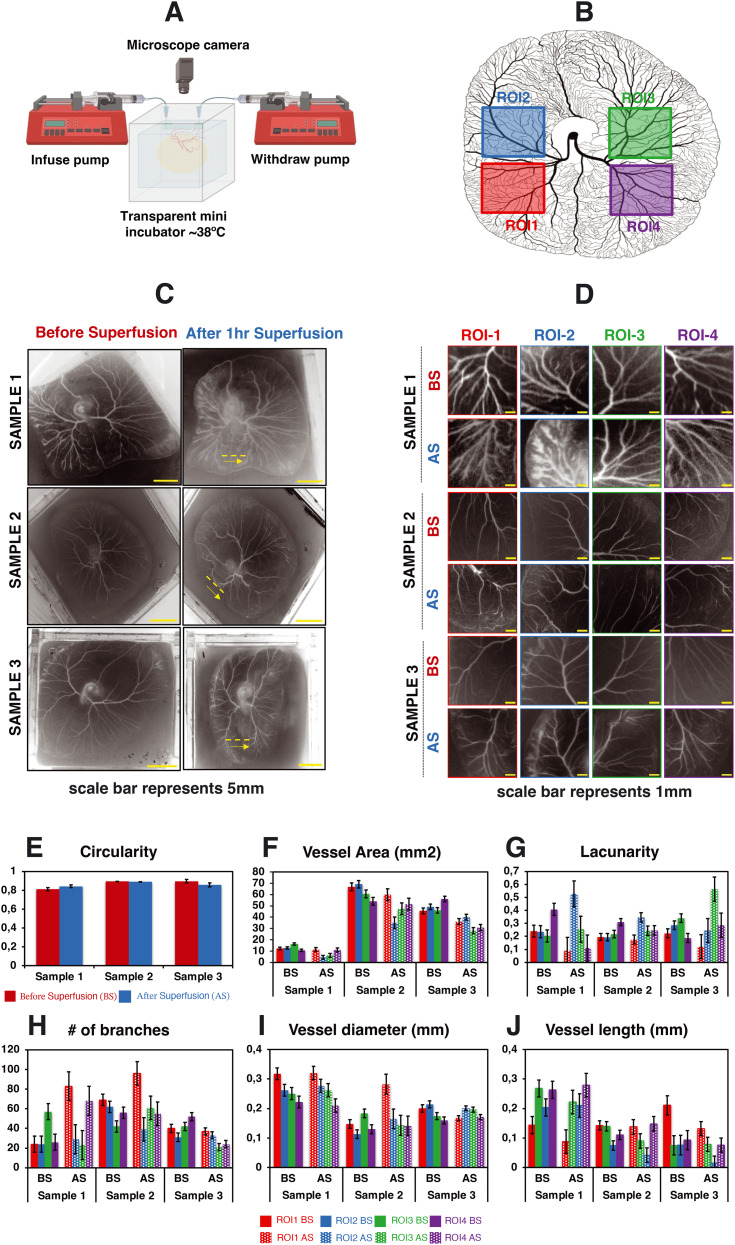
Impact of superfusion on the organization of vascular networks within the CAM. This figure portrays the experimental observations of 3 different test samples. (A) The schematic representation of the perfusion setup. (B) The selected regions of interest for data analysis. (C and D) The spatial distribution of vascular networks of the CAM tissue cultured in the engineered eggshell system before and after the superfusion of PBS at a flow rate of 25 μL min^−1^. Note that the yellow dashed line and arrow line represent the location of microchannel placement and superfused flow direction. (E–J) The quantitative morphometric analysis of the CAM circularity, vessel area, number of branches, lacunarity, vessel length and vessel diameter, respectively; BS = before superfusion, AS = after superfusion. Data analyses were performed using Fiji and Angiotool, and graphs were plotted using Microsoft Excel software application. Statistical analysis test results are included in Fig. S4.†

To study whether superfusion induced fluid flows have an influence on the overall organization of the developing vascular network within the CAM, we carried out superfusion experiments employing a PBS solution. The rationale behind selecting PBS is that it exhibits a viscosity comparable to that of a cell culture medium and has minimal interference with the overall growth of the embryo. Following the integration of the microfluidic flow channel on top of the vascularized CAM, we initiated PBS perfusion at a rate of 25 μl min^−1^ as shown in Videos S3b and S4a and b.†Fig. 4A depicts the schematic representation of the perfusion setup. As our culture platform allows the comprehensive visualization of the vasculature, multiple regions of interest (ROI; see Fig. 4B) were chosen for morphometric analysis.

Based on our computational analysis, flow velocities of albumen/PBS below 0.2 mm s^−1^ are predicted at the level of the CAM vasculature during superfusion. This, in turn, results in a shear stress level of less than 1 dyn cm^−2^, whereas the physiological tissue flow range during primitive streak formation was reported to be between 0.1 and 1.5 μm s^−1^ at day 1 post incubation.^23^ Superfusion experiments were performed on three individual samples at the fourth day of embryonic development (EDD4). Subsequently, microscopy was conducted both before (BS) and after superfusion (AS) of the entire CAM vascularized tissue to evaluate and compare the superfusion effects. Prior to superfusion, the results depict a noticeable increase in both the vasculature area and the number of branching points over time within four ROIs across three individual samples (Fig. 4F and H). Over the same period, there was a distinct decrease in mean lacunarity, attributed to the increased sprouting of new vessel structures (Fig. 4G). An interesting pattern emerged in relation to vessel diameter and length, suggesting an influence on the structural remodeling process (Fig. 4I and J).

Following superfusion, changes were observed in the organization of the vascular networks: a reduction in the vasculature area corresponding to regression, but surprisingly, an increase in branching points. Vessel diameters appeared to follow a similar pattern to that before superfusion, albeit with an increase in size (Fig. 4J). The vessel length exhibited a comparable pattern to the pre-superfusion period (Fig. 4I). The statistical analysis test results are highlighted in Fig. S4.†

**Fig. 5:**
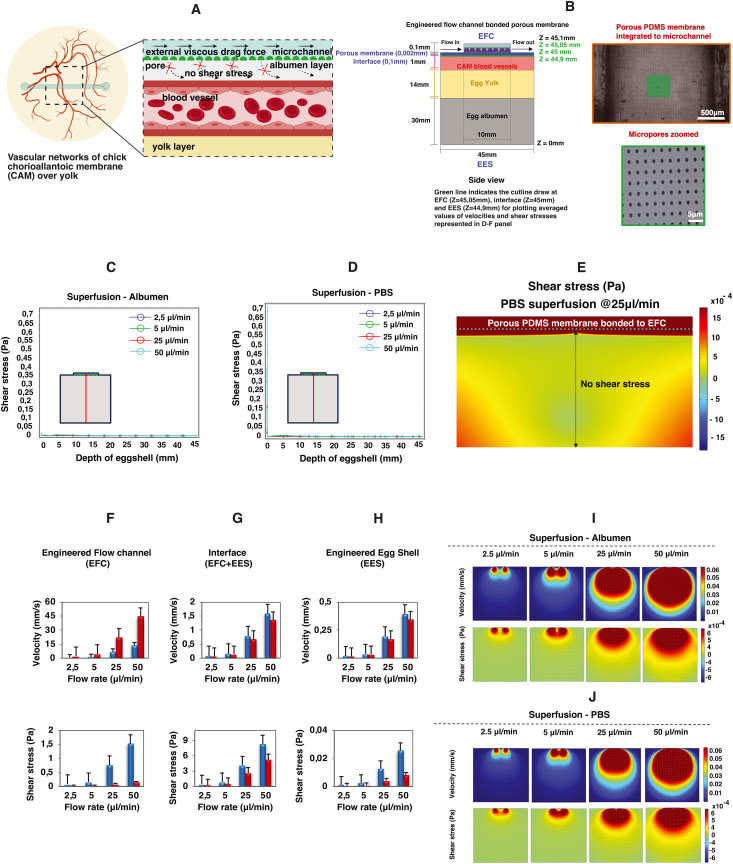
Proof-of-concept study: impact of the porous membrane on superfusion. This figure portrays the 2D simulation data of microfluidic superfusion of an engineered eggshell system with a porous membrane between the perfusion channel and the eggshell. (A) The schematic representation of decreased flow perturbations in the presence of a porous membrane. (B) An illustration of the 2D side view of the engineered eggshell, showcasing the integrated flow channel with a bonded porous membrane serving as the geometrical input for simulation. Note that B shows the bonded porous PDMS membrane integrated to the flow channel. (C and D) The line plot of shear stresses along the depth of the engineered eggshell at increasing flow rates. The red line in the drawing highlights the location of a cut line made in the 2D model for analysis. (E) 2D model and predicted shear stress distribution within the engineered flow channel, interface, and engineered eggshell at the location of CAM blood vessels in the presence of the bonded porous membrane. (F–H) The averaged simulated values of velocities and shear stresses at three specific locations: the middle of the engineered flow channel (EFC) at *Z* = 45.05 mm, the surface of CAM blood vessels within the engineered eggshell (EES) at *Z* = 44.9 mm, and the interface between the EFC and EES at 45 mm from the bottom of the eggshell marked as *Z* = 0 respectively in the presence of the porous membrane. This involves superfusing albumen and PBS fluids through membrane bonded microchannels at progressively increasing flow rates. The three green lines in panel A indicate the locations of the cut lines made in the 2D model for generating the graphs. (I and J) The heatmaps of predicted velocities and shear stresses within the engineered eggshell upon albumen and PBS superfusion at increasing flow rates through the integrated microchannels bonded with the porous membrane. Heatmaps were plotted using COMSOL Multiphysics software version 5.6, and graphs were plotted using Microsoft Excel software application.

The microfluidic flow channel can be used not only for the superfusion of the CAM with the aim of perturbing the mechanical environment of the CAM, but also as a delivery route for chemical perturbations by perfusing, for instance, liquids containing specific growth factors. In order to be able to decouple the chemical perturbation from the mechanical perturbation, we performed a proof-of-concept test where a porous PDMS membrane was included between the microfluidic channel and the engineered eggshell as shown in Fig. 5A and B. Notably, as emphasized in Fig. 5I, J and C–E, it becomes intriguing to observe that flows and shear stresses within the eggshell diminish, with a particular emphasis on the absence of shear stress acting on the CAM blood vessels positioned beneath the porous membrane interface.

To comprehensively explore the contributory roles of fluid flow dynamics in vascular development, we conducted a systematic examination of circulating red blood cells in various vessels located in distinct regions of the vascularized CAM tissue of chick embryos. Our initial focus was on the flows directed toward and away from the heart, as these vessels experienced changes in flow patterns and directionality throughout development and remodeling. Additionally, we recorded the circulating red blood cells within multi-sized vessels of arteries, veins and microcapillaries present within the CAM tissue (Videos S1a and b and S2a–c†). Later, by combining numerical predictions and experimental data, we demonstrate the pivotal role of flow-induced shear stresses in shaping vascular organization throughout development and in response to flow perturbations without and with a porous membrane (Fig. 5 and S3 and Video S5†). This firsthand understanding of shear stress is indispensable for tissue engineers aiming to optimize vascular organization during the perfusion of engineered vascularized tissues.

## Discussion

The development and organization of vascular networks can be influenced in specific spatial ways through genetic, physical, and chemical interventions.^9,10,12,24^ In this context, we introduce the concept of superfusion-induced vascular steering (SIVS) and demonstrate that vascular networks and their associated flow patterns can be adjusted locally through the manipulation of external flows. As far as our knowledge extends, this research represents the first demonstration of applying external flows (superfusion) to vascularized tissues in a live organism. The integration of fluid flow parameters with the configuration of vascular networks advances our comprehensive understanding of structural aspects and mechanical conditions within these developing vascular networks. This holds substantial significance in the regulation of vascular organization.

We engineered an eggshell platform designed to support the development of chick embryos with diverse vascular networks, allowing the integration of microfluidic flow channels on top of the vascularized CAM tissue. This culture platform offers excellent transparency and accessibility from all sides, owing to its cube design, making it ideal for the imaging of the vasculature within the CAM. We recommend our experimental protocol for culturing and transferring the entire egg contents to the engineered eggshell platform (as outlined in Fig. 2A). However, we acknowledge the associated challenges in the transfer of fertilized egg contents to the engineered eggshell system and the placement of microfluidic flow channels in different regions of the vascularized CAM tissue. First, it is essential to emphasize the significance of pre-loading albumen into the engineered eggshell before cracking the native egg. This precaution minimizes potential harm to the vascular structures within the embryo during the cracking process. Secondly, once the entire egg contents have been transferred to the engineered eggshell system, it is important to remove any excess albumen and any air bubbles that may have formed during the transfer. This step helps prevent the microchannel from slipping out of place after placement. Finally, before initiating perfusion, a gentle thumb pressure should be applied to the microfluidic compartment to ensure secure attachment to the surface of the CAM containing the vasculature.

To delineate the connection between vascular organization, blood flows, and shear stresses, we performed imaging of the overall vascular structure both before and after perfusion in the vascularized CAM tissue. Our microscopy data reveal a notable alteration in the arrangement of vascular networks, including an increase in vessel diameter and branching, which corresponds to a slight decrease in vessel length, area, and lacunarity. Numerical predictions affirm a linear relationship, demonstrating that as external perfusion rates increase, superfusion velocities and the associated shear stresses experienced by the vascular structures in the CAM also increase. This study does not elucidate the biological processes that lead to the vascular remodeling as a response to external fluid flow shear stresses. For capillary structures, it is possible that a direct response of endothelial cells to the shear stresses is involved; in larger vessels, the endothelial cells are surrounded by supportive smooth muscle cells, which makes direct response by endothelial cells unlikely. Additionally, the microvessels in the CAM are embedded in a cellularized membrane which will also limit the direct exposure to external fluid flow shear stresses.^25^ This study focuses on the effect of external fluid flow shear stresses. The perfusion of the liquid over the vascularized CAM also results in other factors that can influence vascular organization, such as for instance changes in the concentrations of nutrients, metabolic waste products, or oxygen. Even though it can currently not be ruled out that the vascular remodeling phenomena seen in this study are in part linked to these factors, we believe that the effect of superfusion on the availability of nutrients was limited, given the small volume of the microfluidic channel and the limited time of superfusion. Additionally, the PDMS based artificial eggshell chamber is permeable to oxygen, meaning that oxygen limitations are not to be expected in any of the compartments.

The values for the fluid flow shear stresses that are experienced by the vascularized CAM as modeled in this study rely on the assumption that the height of the superfusion liquid is 100 μm. Although this height is mainly determined by the height of the microfluidic channel, it should be noted that there is no static boundary at the bottom of the perfusion channel. It is therefore possible that pressurization of the system upon perfusion results in a deformation of albumen and therefore an increase of the superfusion liquid height. Although we expect this effect to be limited, especially when using a low-viscosity liquid like PBS for the superfusion, this potential effect should be kept in mind when linking superfusion fluxes to observed vascular remodeling processes. Additionally, the thickness of the layer of albumen over the vascularized CAM will vary between eggs and is experimentally hard to control. This is an additional factor that will affect the actual shear stresses experienced by the vasculature upon superfusion.

Moreover, we showed that the addition of a porous membrane to the flow channel reduces the shear stress along the depth of the engineered eggshell. This holds significant interest for tissue engineers, as it opens the possibility of delivering chemical compounds such as growth factors or drugs without the need for exposure to shear stress.

The rearrangement of vascular networks can be initiated by external flows and specific geometric constraints. These characteristics lead to a diverse network configuration and flow pattern. We propose that such changes in vascular organization may potentially influence locally generated shear stresses within these networks. We speculate that these changes may contribute to shaping the long-range patterning of vascular networks.

## Conclusion

In conclusion, we demonstrate that the introduction of superfusion during vascular development has the potential to alter the overall organization of vascular networks. We attribute these alterations to the accumulation of shear stresses induced by the viscous drag force present in the walls of microfluidic channels located at the top of developing vasculature. Additionally, we highlight the importance of considering both fluid dynamics and structural changes that embryos undergo in their early stages, as these changes impact the circulating blood flow. Our findings offer a fresh perspective on comprehending and testing the impact of microfluidic flows within an *in vivo* environment. We anticipate that the SIVS strategy will inspire further investigations addressing the complexities associated with the incorporation of multidirectional flows within microfluidic chips.

## Materials and methods

### Ethics statement

In accordance with the Dutch animal care guidelines, obtaining IACUC approval for experiments involving chicken embryos is not mandated unless there is an anticipation of hatching. Additionally, IACUC approval is only requisite for experiments involving chick embryos at or beyond EDD14 of development. The embryos utilized in this research were all in the early stages of development, falling between EDD3 and EDD6. The fertilized chicken eggs employed in this study were procured from authorized poultry egg farms in the Netherlands.

### Chick embryo culture

As reported earlier and illustrated in Fig. 2A, Dekalb white fertilized chicken eggs were obtained from Het Anker BV in Ochten, The Netherlands, and stored at 12 °C.^26^ A day before egg transfer, the modified incubator was set to 38 °C with 65% humidity, which was maintained throughout incubation. In the initial 3 day period, the eggs were rotated every two hours for 15 seconds to prevent embryo adhesion to the eggshell. 3 days after starting the incubation, a small ∼2 mm diameter hole was created in the eggshell with fine tweezers, and an 18G syringe microneedle with a plastic syringe was used to withdraw 3 mL of albumen to protect the yolk and embryo vasculature during cracking. Carefully, the withdrawn albumen was introduced into the engineered eggshell to minimize the entry of air bubbles. After this, the chicken embryo containing multi-sized vascular networks were transferred to the engineered eggshell in a sterile environment. The PDMS-based lid containing a flow channel was subsequently positioned onto the CAM blood vessels. Following the careful alignment of the flow channel with blood vessels, thumb pressure was gently applied to ensure a secure attachment.

### Computational fluid dynamics simulations

Computational models were employed to determine the distribution of fluid flow shear stresses within the engineered eggshell and the inner walls of the microfluidic channel. The shear stress acting upon the albumen layer during perfusion was simulated using COMSOL Multiphysics software (version 5.6). The Navier–Stokes equation was solved under laminar flow conditions, with a no-slip boundary condition assumed for the inner walls of the microchannel.

### Perfusion setup

As illustrated in Fig. 4A, the perfusion experiments were conducted under aseptic conditions, employing two syringe pumps (Harvard Apparatus PHD Ultra) connected to the engineered eggshell. One pump was equipped with a sterile syringe (HSW Norm-Ject 25 mL) filled with PBS (Gibco). A sterile 1 mm tube (Nordson Medical Tubing) was affixed to the syringe, with the connection point joined to the microchannel inlet on the engineered eggshell. The second pump contained an empty syringe and was similarly connected to the microchannel outlet. Identical flow and withdrawal rates were employed to ensure consistent control of flow.

### Image acquisition

Images were captured through continuous video recording using a Dino-Lite USB camera (AM4115 ZT 1.3MP) and an HAYEAR digital microscope (16MP Industrial grade, 150× C-Mount Lens). To produce a time lapse video, the recorded video files were processed using the corresponding software: DinoXcope 2.0 for Mac and IC Measure software for Windows. Multiple frames extracted from the processed video were then imported and transformed into 8-bit grayscale images using Fiji software. Vessel morphometrics such as the length, diameter and number of branches were quantified using the Angiotool plugin.

### PDMS porous membrane fabrication

A thin and transparent porous PDMS membrane was bonded to the microchannels to create a barrier between the flow channels and the chick CAM vasculature. As previously detailed, a 2 μm thick, porous PDMS membrane with a 5 μm pore diameter and 30 μm pitch was produced using standard photomask lithography techniques.^27^ Initially, a positive photoresist PR layer (AZ9260, Fujifilm, Japan) was spin-coated onto a 525 μm thick Si wafer (Okmetic, Finland) at 2000 rpm for 60 seconds to achieve a 10 μm thickness. A mask featuring arrays with a 5 μm pore diameter and 30 μm pitch was aligned with the wafer, followed by 17 seconds of UV exposure at an intensity of 12 mW cm^−2^ in hard contact mode. To prevent bubble formation within the thick PR during post-exposure baking, the wafers were left for 1 hour to allow the evaporation of N_2_ gas generated during UV exposure. After this waiting period, the wafers were baked at 120 °C for 2 minutes. A mixture of pre-mixed PDMS (in a 10 : 1 w/w ratio with a curing agent) was diluted with hexane (in a 2 : 5 w/w ratio of PDMS to hexane) to reduce viscosity. Subsequently, the PDMS solution was spin-coated onto the fabricated PR column arrays at 4000 rpm for 1 minute and baked in an oven at 60 °C for at least 3 hours. A plasma etching process was carried out on the cured PDMS membrane using a reactive ion etching system (TEtske, Nanolab University of Twente, the Netherlands) at 47 sccm SF_6_ and 17 sccm O_2_, 100 W, and 50 mTorr for 2 minutes, resulting in a membrane with 2% porosity as shown in the bottom panels in Fig. 5A and B. To transfer the approximately 2 μm thick PDMS membrane to the chip, the inner walls of the microchannel and the porous PDMS surface on the Si wafer underwent oxygen plasma treatment and were brought into contact. To ensure a secure bond, the assembly was heated in an oven at 60 °C for 10 minutes.

## Author contributions

Conceptualization: PP, NSN, and JR; formal analyses: PP; funding acquisition: JR; investigation: PP; methodology: PP and NSN; project administration: PP and JR; software: PP; supervision: JR; validation: PP, NSN, DG, and MZ; visualization: PP and DG; original draft: PP; review and editing: PP, NSN, DG, MZ, LS and JR.

## Conflicts of interest

The authors declare no competing interests.

## Supplementary Material

LC-024-D4LC00311J-s001

LC-024-D4LC00311J-s002
