# Supplementary material for: Switching to external flows: perturbations of developing vasculature within chicken chorioallantoic membrane
Source: Lab Chip. 2024 May 6;24(13):3233–42. doi: 10.1039/d4lc00311j (PMC11198391; doi:10.1039/d4lc00311j)
Supplement: LC-024-D4LC00311J-s001 [file LC-024-D4LC00311J-s001.pdf]

This journal is © The Royal Society of Chemistry 2024

Supplementary Figures

S1. Egg incubator and engineered eggshell design

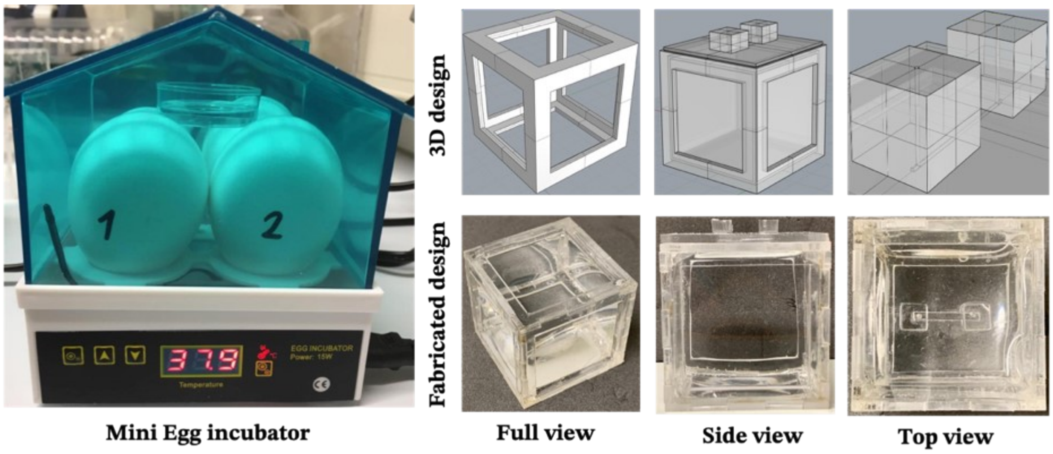

S2. Fertilized egg showing CAM vasculature cultured within engineered eggshell

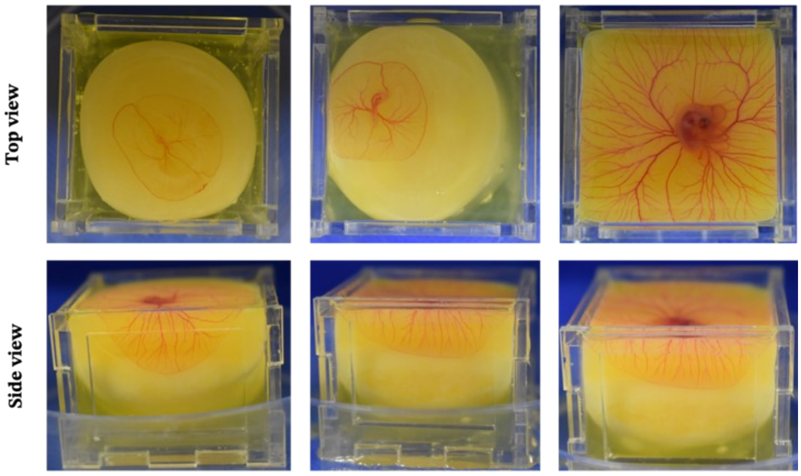

S3. Proof-of-concept test: Impact of superfusion on porous membrane

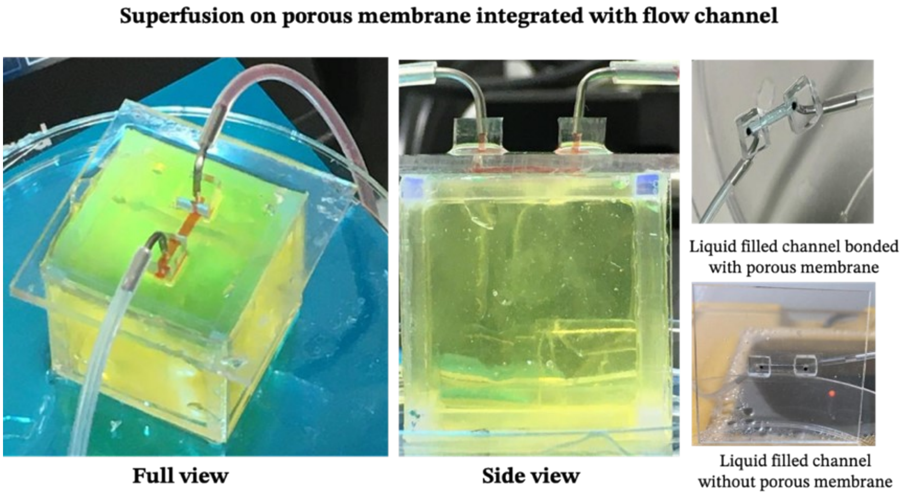

S4. Statistical test

Statistical significance was analyzed using OriginPro V.9.85 performing Wilcoxon signed Rank Test (non-parametric statistical test) due to limited sample numbers. Differences were considered statistically significant with P<0.05. Data shown here are from 3 independent experiment samples, with at least four region of interest (ROIs) per sample.

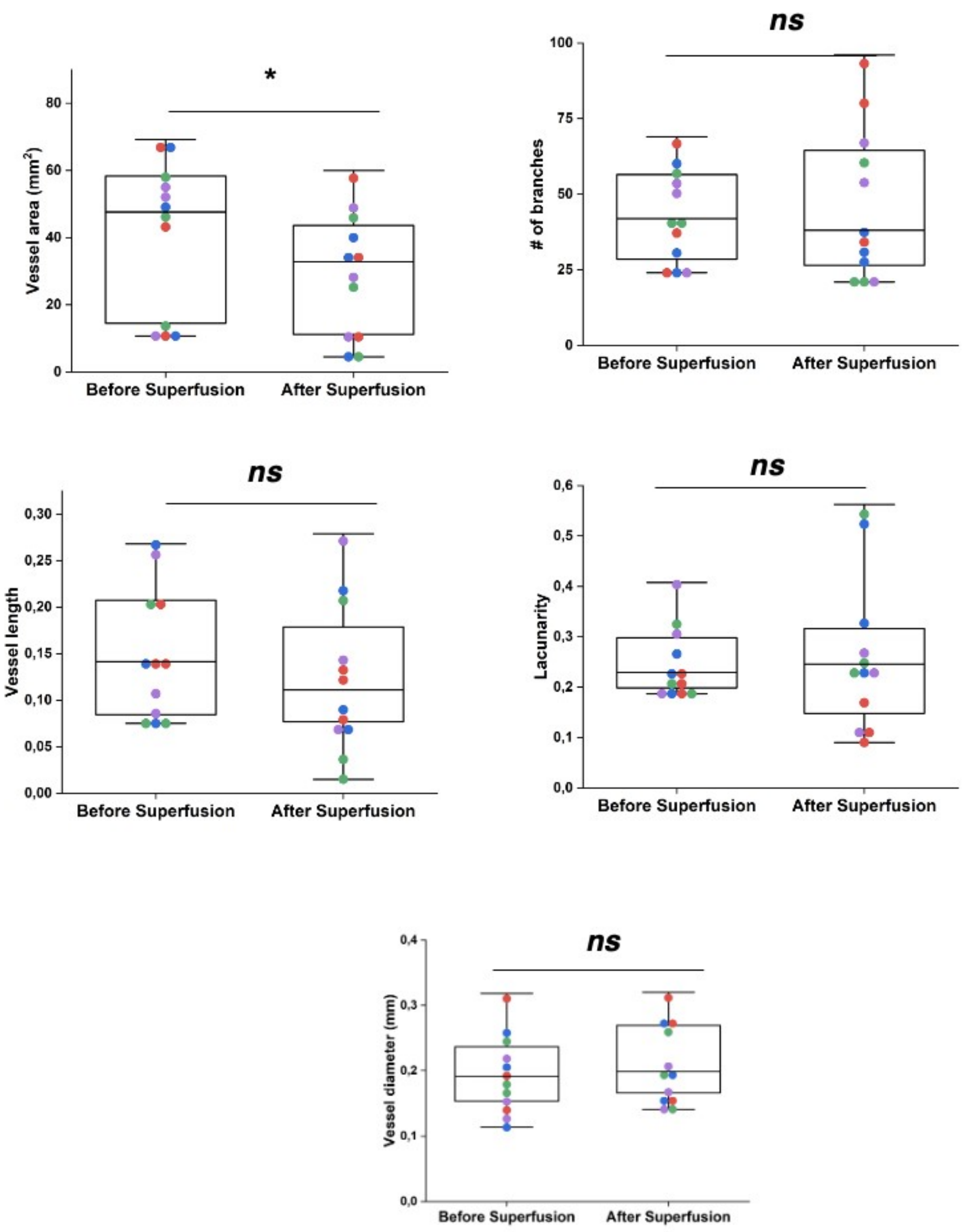

	p- value			
**Morphology parameter**	**S1 (BS vs AS)**	**S2 (BS vs AS)**	**S3 (BS vs AS)**	**All samples (BS vs AS)**
**Vessel area**	0,20124	0,10035	0,10035	0,00326
**Vessel diameter**	0,58388	0,36131	0,85513	0,50474
**Branch number**	0,36131	0,85513	0,20124	0,84452
**Lacunarity**	0,85513	0,85513	0,85513	0,84452
**Vessel length**	0,58388	0,58388	0,20124	0,06526

Supplementary Videos

Supplementary video 1a: Red blood cells flowing through multi-sized venous vessels of vascularized CAM tissue.

Supplementary video 1b: Red blood cells flowing through multi-sized micro capillaries of vascularized CAM tissue.

Supplementary video 2a: Red blood cells flowing through large artery of vascularized CAM tissue.

Supplementary video 2b: Red blood cells flowing through artery-veins of vascularized CAM tissue.

Supplementary video 2c: Red blood cells flowing through artery, microcapillaries and veins of vascularized CAM.

Supplementary video 3a: Chick embryo containing hierarchical vascular networks cultured within engineered eggshell system – chick embryo moving within engineered eggshell system.

Supplementary video 3b: Chick embryo containing hierarchical vascular networks cultured within engineered eggshell system – integrated microchannel perfusion on CAM vasculature.

Supplementary video 4a: External flow application within microchannel integrated on CAM within engineered eggshell system – introducing external fluids via an integrated microchannel.

Supplementary video 4b: External flow application within microchannel integrated on CAM within engineered eggshell system – red blood cells moving within capillaries by external fluid perfusion.

Supplementary video 5: Proof-of-concept testing of integrated porous membrane to microfluidic channels within engineered eggshell system.
